# How does diabetic peripheral neuropathy impact patients' burden of illness and the economy? A retrospective study in Beijing, China

**DOI:** 10.3389/fpubh.2023.1164536

**Published:** 2023-05-12

**Authors:** Qi Pan, Sijia Fei, Lina Zhang, Huan Chen, Jingyi Luo, Weihao Wang, Fei Xiao, Lixin Guo

**Affiliations:** ^1^Department of Endocrinology, Beijing Hospital, National Center of Gerontology, Institute of Geriatric Medicine, Chinese Academy of Medical Sciences, Beijing, China; ^2^Graduate School of Peking Union Medical College, Beijing, China; ^3^The Key Laboratory of Geriatrics, Beijing Institution of Geriatrics, Beijing Hospital, National Center of Gerontology, National Health Commission, Institute of Geriatric Medicine, Chinese Academy of Medical Sciences, Beijing, China

**Keywords:** diabetic peripheral neuropathy (DPN), medications, medical costs, burden of illness, hypoglycemic therapy

## Abstract

**Objective:**

Diabetic peripheral neuropathy (DPN) causes significant illness in patients and has a negative impact on the economy. The objective of this study is to evaluate the cost and quantity of anti-diabetic drugs needed by patients with or without DPN, as well as their variation trends in Beijing between 2016 and 2018.

**Methods:**

This observational cross-sectional study used data on diabetic patients with outpatient medication records obtained from Beijing Medical Insurance from 2016 to 2018. The medications, comorbidities, diabetes-related complications, treatment strategies, and costs of drug treatment were compared between DPN patients and non-DPN patients.

**Results:**

Of the 28,53,036 diabetic patients included in the study, 3,75,216 (13.15%) had DPN and 1,87,710 (50.03%) of the DPN patients were women. Compared with non-DPN patients, DPN patients used more mediations (4.7 ± 2.47 vs. 3.77 ± 2.32, *p* < 0.0001, in 2018) to treat related complications and comorbidities (2.03 ± 1.2 vs. 1.71 ± 1.05; 2.68 ± 1.93 vs. 2.06 ± 1.86, *p* < 0.0001, respectively, in 2018). The total annual costs of drug treatment were higher in DPN patients than in non-DPN patients (¥12583.25 ± 10671.48 vs. ¥9810.91 ± 9234.14, *p* < 0.0001, in 2018). The usage of DDP4i increased from 2.55 to 6.63% in non-DPN patients and from 4.45 to 10.09% in DPN patients from 2017 to 2018.

**Conclusions:**

The number of comorbidities, diabetic complications, medications, and annual drug treatment costs were greater in DPN patients than in non-DPN patients.

## Introduction

Diabetes has become a major public health challenge of the 21st century (1). The 2021 version of the International Diabetes Federation's (IDF) Diabetes Atlas^10^ estimated that one in 10 adults has diabetes. This equals 537 million people (10.5% of the global population) at a global level. The total number of people with diabetes is projected to increase to 643 million (11.3% of the global population) by 2030 and to 783 million (12.2% of the global population) by 2045 (2). This is extremely worrying as an increase in the prevalence of diabetes will also increase the overall number of chronic and acute diseases and have a profound impact on the quality of life, demand for health services, and economic expenses (3). Specifically, diabetes-related comorbidities and complications cause a medical and financial burden, e.g., macrovascular complications of diabetes, including coronary atherosclerotic heart disease (CAD), stroke, and microvascular complications, such as diabetic nephropathy, diabetic retinopathy (DR), and neuropathy, are a significant part of the burden associated with diabetes (3–5).

Extensive literature has quantified the economic burden of diabetes in China (5–7). As reported by the journal Lancet Diabetes & Endocrinology, the global cost of diabetes was US$1.31 trillion, or 1.8% of the global gross domestic product (GDP), in 2015 (5). The World Health Organization (WHO) estimated that the cost of lost productivity for people with diabetes can be more than five times the direct cost of the disease (8). Based on the IDF data, China was ranked first for the number of people with diabetes and the number of people with undiagnosed diabetes, as well as second in the world for diabetes-related health spending (2).

As the prevalence of diabetes increases, the burden of diabetes-related complications is set to increase as well. Diabetic peripheral neuropathy (DPN) is one of the most common chronic complications of diabetes, and it is estimated that it occurs in up to half of all individuals with type 1 or type 2 diabetes mellitus (9, 10). The first China Diabetes Atlas showed that the prevalence of DPN among type 2 diabetes mellitus (T2DM) in China fluctuated between 8.4 and 61.8% (11). The prevalence of comorbidities, such as hypertension, dyslipidemia, CAD, and osteoporosis is higher in diabetic patients with DPN than in those without DPN (12–14). In general, DPN also imposed a heavy financial burden on the healthcare system and society (15). It was reported that a quarter of the US healthcare expenditure on diabetes was spent on DPN (16, 17). In Europe, the direct cost of amputation per patient varied from US $13,842 in 2001 to US $83,728 between 2005 and 2009 (18). However, there is a lack of research on the overall healthcare costs and costs of anti-diabetic drugs for diabetes patients with DPN in China.

In this study, we conducted a cost-effectiveness analysis to examine the differences in glycemic control strategies and healthcare costs between diabetic patients with DPN and those without DPN and to investigate changes in their glucose-lowering medications and related costs, using medical insurance data from 2016 to 2018 in Beijing, China.

## Patients and methods

### Study design and setting

We conducted a retrospective, observational study to analyze the differences in anti-diabetic medication and related costs in diabetes patients with and without DPN. The study was approved by the Ethics Committee of the Beijing Hospital.

### Study participants and data collection

This study enrolled diabetic patients who had outpatient medical records with Beijing Medical Insurance from 2016 to 2018. The diagnosis of diabetes is based on the World Health Organization's (WHO) 1999 criteria. The study's exclusion criteria were (1) no diagnosis of diabetes (no diabetes recorded in the primary or secondary diagnosis) and (2) no continuous prescription recorded for more than 2 months. In the current health insurance reimbursement system, it is not possible to prescribe drugs for more than 30 days at a time.

We collected the following information from the Beijing health insurance database: ICD diagnosis, gender, age, comorbidities, complications, medications (hypoglycemic and non-hypoglycemic), insulin use, medication costs, etc.

### Definitions of comorbidities and complications

We divided diabetic patients into DPN and non-DPN groups based on outpatient prescriptions in the Beijing medical insurance database.

Comorbidities in people with diabetes include hypertension, CAD, dyslipidemia, chronic respiratory disease (CRD), and osteoporosis. Diabetes-related complications are as follows: DPN, diabetic kidney disease (DKD), DR, and diabetic angiopathy (DA).

### Anti-glycemic treatment and definitions used

Receiving hypoglycemic medication was defined as the patient having received at least one hypoglycemic drug therapy in at least one of the study years. In this study, the glucose-lowering drugs covered by medical insurance included insulin and oral hypoglycemic agents (OADs). OADs contain α-glucosidase inhibitors (AGIs), metformin, sulfonylureas (SUs), thiazolidinediones, and glinides. Types of insulin include rapid, short-, medium-, and long-term actions, as well as premixed insulin. We classified diabetes treatment strategies into the following three categories: (1) monotherapy: patients who have received only one documented prescription of a hypoglycemic drug in the past year, (2) oral combination therapy: patients taking two or more different classes of oral hypoglycemic drugs within the past year, and (3) combined OADs and insulin therapy: the patient has had at least one prescription of an OAD and one prescription of insulin in the past year.

### Statistical analysis

The study data were statistically analyzed using SAS software version 9.4. Data satisfying a normal distribution were shown as mean ± standard deviation (x ± SD), such as the number of medications, comorbidities, medication costs, and percentage distribution of diagnosed diseases, such as hypertension, dyslipidemia, and osteoporosis. These data were compared statistically using the *Wilcoxon rank sum test*. Negative binomial models and log link functions were used when the distribution of variables was too dispersed. The *multivariate regression model* was used to control for confounders. Data on categorical variables were expressed as numbers and percentages, such as the insulin usage rate and the gender/age ratio. A *chi-square test* (χ^2^) and *Fisher's exact test* were used for comparison; a *p*-value of < 0.05 was considered statistically significant.

## Results

### Baseline characteristics

**Figure 1:**
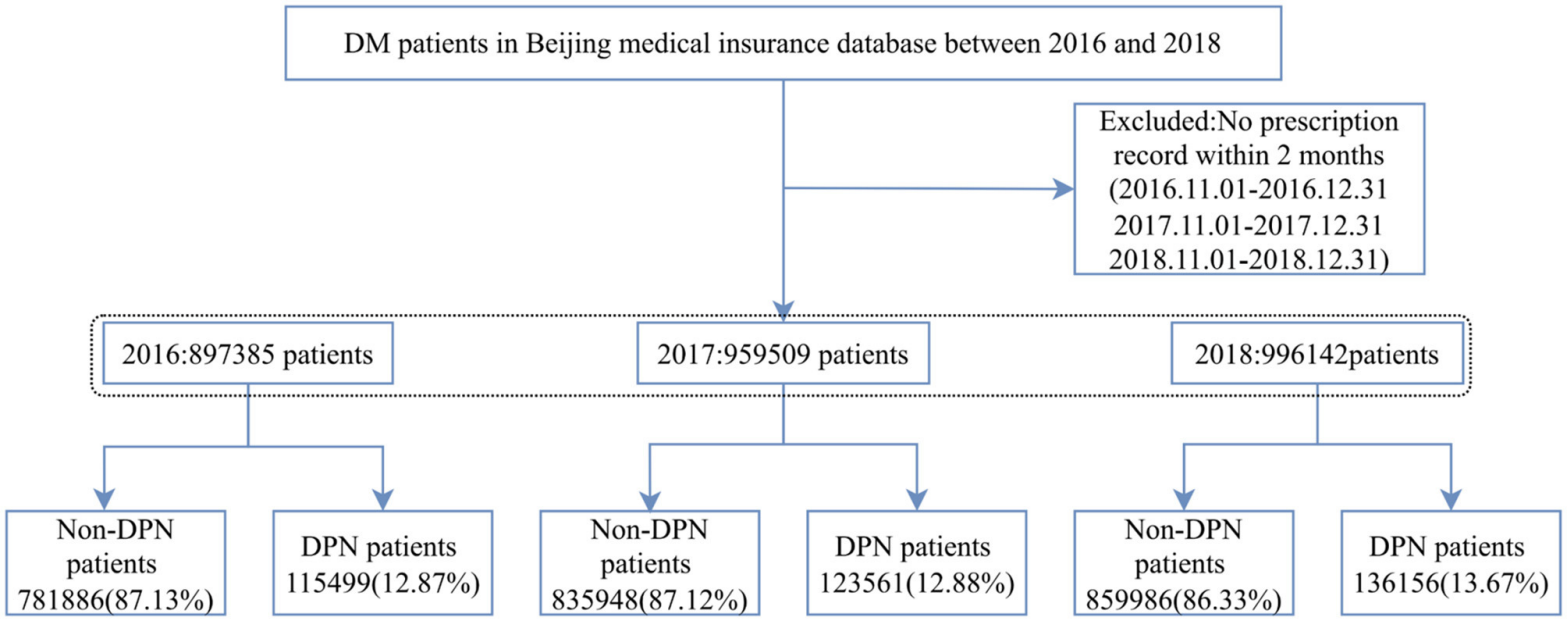
Patient enrollment flow chart. DM, diabetes mellitus; DPN, diabetic peripheral neuropathy.

In total, 28,53,036 diabetic patients were enrolled in this study (8,97,385 diabetic patients in 2016, 9,59,509 diabetic patients in 2017, and 9,96,142 diabetic patients in 2018). A flowchart of patient enrollment is shown in Figure 1. Of those, 3,75,216 (13.15%) had DPN and 1,87,710 (50.03%) of DPN patients were women (Table 1).

**Table 1:** Numbers of medications in stratified patient groups.

	**Non-DPN**				**DPN**				**Wilcoxon test *p*-value**
	***N***	**Mean**	**Adjusted mean**	**Sd**	***N***	**Mean**	**adjusted mean**	**Sd**	
**Age**									
15–44 y	2,16,630	2.65	2.64	2.02	17,565	3.68	3.68	2.31	< 0.0001
45–64 y	13,15,710	3.69	3.69	2.3	1,74,950	4.62	4.62	2.46	< 0.0001
65–84 y	8,82,916	3.98	3.98	2.37	1,71,201	4.8	4.80	2.5	< 0.0001
≥85 y	62,564	3.85	3.85	2.31	11,500	4.41	4.42	2.49	< 0.0001
**Gender**									
Male	1,276,872	3.77	3.47	2.33	1,87,506	4.68	4.24	2.46	< 0.0001
Female	12,00,948	3.64	4.41	2.33	1,87,710	4.62	5.16	2.5	< 0.0001
**Hypertension**									
	14,97,645	4.74	4.43	2.19	2,58,791	5.48	4.90	2.31	< 0.0001
CAD									
	11,87,407	4.83	2.70	2.28	2,23,157	5.44	3.02	2.41	< 0.0001
**Dyslipidemia**									
	11,06,492	4.95	5.14	2.28	2,22,411	5.58	5.50	2.32	< 0.0001
**CRD**									
	4,42,464	4.25	6.17	2.36	85,821	5.14	6.55	2.54	< 0.0001
**Osteoporosis**									
	3,22,964	4.42	4.18	2.33	83,257	5.08	4.88	2.49	< 0.0001
**DPN**									
					3,75,216	4.65	5.52	2.48	
**DKD**									
	92,672	4.51	3.57	2.57	28,077	5.56	4.40	2.52	< 0.0001
**DR**									
	75,372	4.72	2.07	2.61	51,655	5.51	2.71	2.52	< 0.0001
**DA**									
	43,537	4.8	2.60	2.48	45,822	5.24	3.35	2.48	< 0.0001
**Number of comorbidities**									
0	4,80,262	1.5	1.48	1.1	37,038	1.86	1.80	1.35	< 0.0001
1	5,51,143	2.75	2.70	1.56	63,274	3.12	3.02	1.74	< 0.0001
2	6,04,051	3.91	3.83	1.87	92,854	4.35	4.22	1.99	0.201
3	6,01,221	5.27	5.14	2.14	1,14,730	5.66	5.50	2.23	< 0.0001
4	2,11,651	5.85	5.72	2.17	56,335	6.26	6.10	2.29	< 0.0001
5	29,492	6.29	6.17	2.26	10,985	6.71	6.55	2.42	< 0.0001
**Number of complications**									
0	22,83,312	3.64	3.43	2.29					
1	1,78,188	4.47	4.18	2.55	2,68,837	4.39	4.12	2.42	< 0.0001
2	15,567	5.53	5.15	2.46	88,519	5.21	4.88	2.5	< 0.0001
3	753	5.97	5.52	2.51	16,545	5.88	5.51	2.46	< 0.0001
4					1,315	6.42	6.02	2.48	

The multivariable regression model included the covariables: age, gender, hypertension, CAD, dyslipidemia, CRD, osteoporosis, DPN, DKD, DR, DA, and year; 0, absence of the disease; 1, presence of the disease; CAD, coronary atherosclerotic heart disease; DA, diabetic angiopathies; DKD, diabetic kidney disease; DPN, diabetic peripheral neuropathy; DR, diabetic retinopathy; 0, 1, 2, 3 ,4, 5, the number of comorbidities or complications.

The age of the diabetic patients in this study was mostly concentrated in the age group of 45–84 years, accounting for 89.2% (25,44,777/28,53,036). In addition, 51.3% of the diabetic patients in this study were men (14,64,378/28,53,036). Among the patients with DPN included in this study, the age of onset was mostly concentrated in the age group of 45–84 years, accounting for 92.25% (3,46,151/3,75,216) (Table 2).

**Table 2:** Demographic characteristics of diabetic patients in Beijing between 2016 and 2018.

	**Non-DPN**			**DPN**			**χ^2^ test 2016, *p* value**	**χ^2^ test 2017, *p* value**	**χ^2^ test 2018, *p* value**
	**2016 (100%)**	**2017 (100%)**	**2018 (100%)**	**2016 (100%)**	**2017 (100%)**	**2018 (100%)**			
Total	7,81,886 100.00%	8,35,948 100.00%	8,59,986 100.00%	1,15,499 100.00%	1,23,561 100.00%	1,36,156 100.00%	< 0.0001	< 0.0001	< 0.0001
**Age group**									
15–44 y	68,496 (8.8%)	73,587 (8.8%)	74,547 (8.7%)	5,647 (4.9%)	5,858 (4.7%)	6,060 (4.5%)			
45–64 y	423,252 (54.1%)	4,44,728 (53.2%)	4,47,730 (52.1%)	55,396 (48%)	57,521 (46.6%)	62,033 (45.6%)			
65–84 y	2,72,710 (34.9%)	2,96,803 (35.5%)	3,13,403 (36.4%)	51,453 (44.5%)	56,370 (45.6%)	63,378 (46.5%)			
≥85 y	17,428 (2.2%)	20,830 (2.5%)	24,306 (2.8%)	3,003 (2.6%)	3,812 (3.1%)	4,685 (3.4%)			
**Gender (%)**							< 0.0001	< 0.0001	< 0.0001
Male	3,94,568 (50.5%)	4,29,064 (51.3%)	4,53,240 (52.7%)	56,858 (49.2%)	61,827 (50%)	68,821 (50.5%)			
Female	3,87,318 (49.5%)	4,06,884 (48.7%)	4,06,746 (47.3%)	58,641 (50.8%)	61,734 (50%)	67,335 (49.5%)			
**Hypertension (%)**							< 0.0001	< 0.0001	< 0.0001
	4,70,879 (60.2%)	5,09,001 (60.9%)	5,17,765 (60.2%)	79,719 (69%)	85,910 (69.5%)	93,162 (68.4%)			
**CAD (%)**							< 0.0001	< 0.0001	< 0.0001
	3,72,711 (47.7%)	4,08,205 (48.8%)	4,06,491 (47.3%)	68,726 (59.5%)	74,600 (60.4%)	79,831 (58.6%)			
**Dislipidemia (%)**							< 0.0001	< 0.0001	< 0.0001
	3,36,375 (43%)	3,76,701 (45.1%)	3,93,416 (45.7%)	66,695 (57.7%)	73,828 (59.8%)	818,88 (60.1%)			
**CRD (%)**							< 0.0001	< 0.0001	< 0.0001
	1,40,807 (18%)	1,55,560 (18.6%)	1,46,097 (17.0%)	26,834 (23.2%)	29,395 (23.8%)	29,592 (21.7%)			
**Osteoporosis (%)**							< 0.0001	< 0.0001	< 0.0001
	1,06,274 (13.6%)	1,12,640 (13.5%)	1,04,050 (12.1%)	26,294 (22.8%)	28,034 (22.7%)	28,929 (21.2%)			
**DKD**							< 0.0001	< 0.0001	< 0.0001
	33,221 (4.2%)	31,445 (3.8%)	28,006 (3.3%)	9,858 (8.5%)	9,334 (7.6%)	8,885 (6.5%)			
**DR**							< 0.0001	< 0.0001	< 0.0001
	25,282 (3.2%)	25,952 (3.1%)	24,138 (2.8%)	16,615 (14.4%)	17,437 (14.1%)	17,603 (12.9%)			
**DA**							< 0.0001	< 0.0001	< 0.0001
	14,494 (1.9%)	14,762 (1.8%)	14,281 (1.7%)	14,751 (12.8%)	15,098 (12.2%)	15,973 (11.7%)			

CAD, coronary atherosclerotic heart disease; CRD, chronic respiratory disease; DA, diabetic angiopathies; DKD, diabetic kidney disease; DPN, diabetic peripheral neuropathy; DR, diabetic retinopathy.

We compared DPN patients with non-DPN patients in terms of comorbidities and complications. DPN patients were more likely to have comorbidities, such as hypertension (68.4 vs. 60.2%, *p* < 0.0001), CHD (58.6 vs. 47.3%, *p* < 0.0001), dyslipidemia (60.1 vs. 45.7%, *p* < 0.0001), chronic respiratory disease (CRD) (21.7 vs. 17.0%, *p* < 0.0001), and osteoporosis (21.2 vs. 12.1%, *p* < 0.0001) than those without DPN. Diabetic patients with DPN were also more likely to develop complications, such as DKD (6.5 vs. 3.3%, *p* < 0.0001), DR (12.9 vs. 2.8%, *p* < 0.0001), and DA (11.7 vs. 1.7%, *p* < 0.0001) than those without DPN (all *p*'s < 0.0001 from 2016 to 2018; we only list data from 2018 in the text) (Table 1).

### Differences in medications and costs between diabetic patients with DPN and without DPN

We further revealed that diabetic patients with DPN are more likely to develop comorbidities than non-DPN patients (3.7 ± 1.46 vs. 1.91 ± 1.38, *p* < 0.0001), both in terms of glycemic diseases and non-glycemic diseases (2.03 ± 1.2 vs. 1.71 ± 1.05; 2.68 ± 1.93 vs. 2.06 ± 1.86, *p* < 0.0001, respectively) (Table 3). Then, we focused on medications and the costs of treating diabetic patients. DPN patients used more mediations than non-DPN patients (4.7 ± 2.47 vs. 3.77 ± 2.32, *p* < 0.0001), including hypoglycemic drugs and non-hypoglycemic drugs (2.03 ± 1.2 vs. 1.71 ± 1.05; 2.68 ± 1.93 vs. 2.06 ± 1.86, *p* < 0.0001, respectively). In terms of medication costs, DPN patients had higher costs than non-DPN patients (¥12583.25 ± 10671.48 vs. ¥9810.91 ± 9234.14, *p* < 0.0001), including the cost of hypoglycemic drugs and non-hypoglycemic drugs (¥6497.28 ± 8190.2 vs. ¥5138.49 ± 6791.55; ¥6085.97 ±5 946.58 vs. ¥4672.43 ± 5547.26, *p* < 0.0001, respectively). Meanwhile, we found that there was a significant difference between the total annual drug cost/number of drugs between DPN patients and non-DPN patients, which was higher in DPN patients (2613.24 ± 2003.41 vs. 2520.95 ± 2509.1, *p* < 0.0001), not only regarding the total annual hypoglycemic drug cost/number of drugs but also regarding non-hypoglycemic drugs (¥2800.55 ± 3067.12 vs. ¥2634.51 ± 3216.73; ¥1987.74 ± 1751.53 vs. ¥1716.11 ± 1919.47, *p* < 0.0001, respectively) (all *p*'s < 0.0001 from 2016 to 2018; we only listed data from 2018 in the text) (Table 3).

**Table 3:** The difference in medications, comorbidities, and drug costs between non-DPN and DPN diabetes patients.

	**Non-DPN**			**DPN**			**2016, *p-*value**	**2017, *p-*value**	**2018, *p-*value**
	**2016**	**2017**	**2018**	**2016**	**2017**	**2018**			
Number of medications	3.61 ± 2.32	3.74 ± 2.35	3.77 ± 2.32	4.56 ± 2.48	4.69 ± 2.5	4.7 ± 2.47	< 0.0001	< 0.0001	< 0.0001
Hypoglycemic drugs	1.57 ± 1.02	1.65 ± 1.04	1.71 ± 1.05	1.88 ± 1.16	1.97 ± 1.19	2.03 ± 1.2	< 0.0001	< 0.0001	< 0.0001
Non-hypoglycemic drugs	2.04 ± 1.87	2.09 ± 1.88	2.06 ± 1.86	2.68 ± 1.95	2.73 ± 1.95	2.68 ± 1.93	< 0.0001	< 0.0001	< 0.0001
Number of comorbidities	1.94 ± 1.42	1.97 ± 1.41	1.91 ± 1.38	3.79 ± 1.51	3.79 ± 1.5	3.7 ± 1.46	< 0.0001	< 0.0001	< 0.0001
Glycemic diseases	0.09 ± 0.32	0.09 ± 0.31	0.08 ± 0.29	1.36 ± 0.59	1.34 ± 0.58	1.31 ± 0.56	< 0.0001	< 0.0001	< 0.0001
Non-glycemic diseases	1.83 ± 1.3	1.87 ± 1.3	1.82 ± 1.28	2.32 ± 1.29	2.36 ± 1.28	2.3 ± 1.26	< 0.0001	< 0.0001	< 0.0001
Total annual cost/drug^¥^	10609.56 ± 10697.11	9711.13 ± 9530.32	9810.91 ± 9234.14	13933 ± 12331.66	12497.54 ± 10998.16	12583.25 ± 10671.48	< 0.0001	< 0.0001	< 0.0001
Hypoglycemic drugs	5333.88 ± 7757.33	4984.59 ± 6896.31	5138.49 ± 6791.55	6927 ± 9179.59	6333.8 ± 8260.16	6497.28 ± 8190.2	< 0.0001	< 0.0001	< 0.0001
Non-hypoglycemic drugs	5275.68 ± 6525.57	4726.54 ± 5772.07	4672.43 ± 5547.26	7005.99 ± 7193.24	6163.74 ± 6278.65	6085.97 ± 5946.58	< 0.0001	< 0.0001	< 0.0001
Cost/drug^¥^	2821.75 ± 3016.4	2494.36 ± 2443.6	2520.95 ± 2509.1	2975.65 ± 2528.02	2588.96 ± 2075.87	2613.24 ± 2003.41	< 0.0001	< 0.0001	< 0.0001
Cost/anti-glycemic drug	2900.98 ± 3819.06	2609.75 ± 3234.16	2634.51 ± 3216.73	3158.28 ± 3996.46	2788.41 ± 3399.56	2800.55 ± 3067.12	< 0.0001	< 0.0001	< 0.0001
Cost/non-anti-glycemic drug	1928.28 ± 2303.26	1703.68 ± 1959.39	1716.11 ± 1919.47	2256.86 ± 2077.67	1967.46 ± 1842.59	1987.74 ± 1751.53	< 0.0001	< 0.0001	< 0.0001

DPN, diabetic peripheral neuropathy. ^¥^: Chinese Yuan.

Diabetic patients with DPN used more drugs than non-DPN patients regardless of their age, gender, or whether they had hypertension, dyslipidemia, CHD, osteoporosis, DKD, DR, or DA. In addition, the number of drugs used increases with the number of comorbidities or complications (Table 3). Similarly, regardless of age, gender, comorbidities, or complications of diabetes, diabetic patients had higher total annual drug costs. As the number of comorbidities or complications increases, the cost of medication also increases (Table 4).

**Table 4:** Total annual drugs costs of non-DPN and DPN diabetes patients.

	**Non-DPN**				**DPN**				**Wilcoxon test** ***p-*****value**	
	***N***	**Mean**	**Adjusted mean**	**SD**	***N***	**Mean**	**Adjusted mean**	**Sd**		
**Age**										
15–44 y	2,16,630	7517.8	7495.06	9323.67	17,565	11010.88	10989.55	12020.98	58.8546	< 0.0001
45–64 y	13,15,710	10000.89	9996.60	9810.73	1,74,950	12951.69	12946.75	11477.49	96.4691	< 0.0001
65–84 y	8,82,916	10644.98	10643.45	9840.9	1,71,201	13244.19	13250.62	11099.43	101.6165	< 0.0001
≥85 y	62,564	10632.87	10636.00	10127.42	11,500	12175.5	12181.82	11059.09	105.0678	< 0.0001
**Sex**										
Male	1,276,872	10128.95	9504.30	9952.88	1,87,506	13056.3	11911.98	11280.53	178.0398	< 0.0001
Female	12,00,948	9923.28	12728.51	9683.06	1,87,710	12884.8	14978.93	11379.52	92.8037	< 0.0001
Hypertension	14,97,645	12330.72	12515.21	10045.48	2,58,791	14856.5	14194.36	11610.92	64.2892	< 0.0001
CAD	11,87,407	13161.26	7127.39	10302.23	223157	15196.79	8445.59	11906.89	142.5171	< 0.0001
Dyslipidemia	11,06,492	13509.67	13976.04	10404.05	2,22,411	15669.63	15257.10	11739.75	173.6331	< 0.0001
CRD	4,42,464	11800.09	18571.81	10445.18	85,821	14975.58	20339.09	12428.67	182.3109	< 0.0001
Osteoporosis	3,22,964	12252.4	12483.96	10612.66	83,257	14493.08	14245.55	12189.42	40.4147	< 0.0001
DPN					3,75,216	12970.5	16707.28	11330.47		
DKD	92,672	14135.44	9736.14	12460.96	28,077	17338.63	12417.97	13699.57	61.5765	< 0.0001
DR	75,372	13372.6	6404.78	11583.21	51,655	15752.66	8464.19	12124.82	30.3318	< 0.0001
DA	43,537	13284.39	7017.06	11693.37	45,822	14963.77	9367.49	12106.85	14.0058	< 0.0001
**Number of comorbidities**										
0	4,80,262	4760.23	4727.26	8037.12	37,038	5945.46	5769.27	8385.7	36.1948	< 0.0001
1	5,51,143	7174	7127.39	7538.94	63,274	8643.61	8445.59	8667.65	17.7083	< 0.0001
2	6,04,051	10152.27	10090.00	8521.55	92,854	11741.67	11524.15	9630.3	2.0027	0.0226
3	6,01,221	14069.36	13976.044	10256.52	1,14,730	15485.85	15257.10	11336.9	17.9445	< 0.0001
4	2,11,651	16398.58	16337.20	11223.03	56,335	17890.62	17706.73	12905.61	147.8046	< 0.0001
5	29,492	18601.52	18571.81	12485	10,985	20463.85	20399.09	14194.39	145.146	< 0.0001
**Number of complications**										
0	22,83,312	9740.12	9342.23	9576.2						
1	1,78,188	13128.78	12483.96	11652.9	2,68,837	11993.89	11424.32	10775.11	115.0047	< 0.0001
2	15,567	16569.34	15750.07	13429.17	88,519	14970.68	14245.55	12016.59	139.7284	< 0.0001
3	753	18140.7	17169.68	11885.64	16,545	17555.11	16707.28	12976.86	141.8745	< 0.0001
4					1,315	20304.79	19327.80	16560.8		

The multivariable regression model included the covariables: age, gender, hypertension, CAD, dyslipidemia, CRD, osteoporosis, DPN, DKD, DR, DA, and year; 0, absence of the disease; 1, the presence of the disease; CAD, coronary atherosclerotic heart disease; DA, diabetic angiopathies; DKD, diabetic kidney disease; DPN, diabetic peripheral neuropathy; DR, diabetic retinopathy; 0, 1, 2, 3, 4, 5, the number of comorbidities or complications.

### The difference in diabetes therapy regimens between DPN and non-DPN patients

Diabetic patients preferred using premixed insulin. In 2016–2018, the proportions of diabetic patients with DPN using premixed insulin were 55.5, 52.8, and 50.3%, respectively; and the proportions of diabetic patients without DPN using premixed insulin were 61.6, 59.8, and 56.3%, respectively. Regardless of whether patients were DPN or non-DPN patients, the usage of premixed, short-acting, and intermediate-acting insulins decreased each year during 2016–2018, and the use of fast-acting and long-acting insulins gradually increased.

Compared to non-DPN diabetic patients, diabetic patients with DPN were less likely to use premixed insulin (55.5 vs. 61.6%, *p* < 0.0001, in 2016; 52.8 vs. 59.8%, *p* < 0 .0001, in 2017; 50.3 vs. 56.3%, *p* < 0.0001, in 2018), more likely to use fast-acting insulin (11.4 vs. 7.5%, *p* < 0.0001, in 2016; 13.5 vs. 9.5%, *p* < 0.0001, in 2017; 14.7 vs. 11.2%, *p* < 0.0001, in 2018), more likely to use intermediate-acting insulin (16.3 vs. 15.1%, *p* < 0.001; 14.6 vs. 13.5%, *p* < 0.0001; 13.0 vs. 12.2%, *p* < 0.0001), and more likely to use long-acting insulin (26.1 vs. 19.7%, *p* < 0.001; 30.6 vs. 23.2%, *p* < 0.0001; 34.3 vs. 27.6%, *p* < 0.0001, in 2018) (Table 5).

**Table 5:** Different types of insulin used by non-DPN and DPN diabetes patients from 2016 to 2018.

**Type of insulin**	**Non-DPN**			**DPN**			**2016, *p*-value**	**2017, *p-*value**	**2018, *p-*value**
	**2016**	**2017**	**2018**	**2016**	**2017**	**2018**			
**Fast-acting**							< 0.0001	< 0.0001	< 0.0001
0	1,83,976 (92.5%)	1,90,225 (90.5%)	1,84,453 (88.8%)	38,460 (88.6%)	39,289 (86.5%)	41,466 (85.3%)			
1	14,980 (7.5%)	19,892 (9.5%)	23,372 (11.2%)	4,926 (11.4%)	6,157 (13.5%)	7,125 (14.7%)			
**Short-acting**							0.0003	< 0.0001	0.1389
0	1,73,647 (87.3%)	1,86,014 (88.5%)	1,85,793 (89.4%)	37,586 (86.6%)	39,925 (87.9%)	43,328 (89.2%)			
1	25,309 (12.7%)	24,103 (11.5%)	22,032 (10.6%)	5,800 (13.4%)	5,521 (12.1%)	5,263 (10.8%)			
**Intermediate-acting**							< 0.0001	< 0.0001	< 0.0001
0	1,68,896 (84.9%)	1,81,755 (86.5%)	1,82,437 (87.8%)	36,326 (83.7%)	38,797 (85.4%)	42,257 (87.0%)			
1	30,060 (15.1%)	28,362 (13.5%)	25,388 (12.2%)	7,060 (16.3%)	6,649 (14.6%)	6,334 (13.0%)			
**Long-acting**							< 0.0001	< 0.0001	< 0.0001
0	1,59,763 (80.3%)	1,61,271 (76.8%)	1,50,533 (72.4%)	32,066 (73.9%)	31,522 (69.4%)	31,928 (65.7%)			
1	39,193 (19.7%)	48,846 (23.2%)	57,292 (27.6%)	11,320 (26.1%)	13,924 (30.6%)	16,663 (34.3%)			
**Premixed**							< 0.0001	< 0.0001	< 0.0001
0	76,378 (38.4%)	84,540 (40.2%)	90,909 (43.7%)	19,328 (44.5%)	21,437 (47.2%)	24,166 (49.7%)			
1	1,22,578 (61.6%)	1,25,577 (59.8%)	1,16,916 (56.3%)	24,058 (55.5%)	24,009 (52.8%)	24,425 (50.3%)			

DPN, diabetic peripheral neuropathy.

In this study, the vast majority of diabetic patients with or without DPN initiated glucose-lowering drug therapy (86.2–89.3%). DPN patients preferred OAD combination therapy (52.6 vs. 44.9%, *p* < 0.0001, in 2018) and preferred a combination of three OADs (AGIs + Metformin+ SUs) (6.2 vs. 5.3%, *p* < 0.001, in 2018). Patients with DPN also preferred OADs in combination with insulin therapy (29.3 vs. 17.5%, *p* < 0.0001) (Table 6).

**Table 6:** Differences in therapy regimens between non-DPN and DPN diabetes patients from 2016 to 2018.

		**Non-DPN**			**DPN**			**2016, *p-*value**	**2017, *p-*value**	**2018, *p-*value**
**2016 (%)**	**2017 (%)**	**2018 (%)**	**2016 (%)**	**2017 (%)**	**2018 (%)**			
**Receiving any antidiabetic drugs**								< 0.0001	< 0.0001	< 0.0001
	0	1,07,836 (13.8%)	1,04,007 (12.4%)	95,037 (11.1%)	15,051 (13.0%)	14,645 (11.9%)	14514 (10.7%)			
	1	6,74,050 (86.2%)	7,31,941 (87.6%)	7,64,949 (88.9%)	1,00,448 (87%)	1,08,916 (88.1%)	121642 (89.3%)			
**Monotherapy**								< 0.0001	< 0.0001	< 0.0001
		2,84,280 (36.4%)	2,90,545 (34.8%)	2,92,701 (34.0%)	27,600 (23.9%)	28,259 (22.9%)	30,950 (22.7%)			
α**-glucosidase**								< 0.0001	< 0.0001	< 0.0001
		1,02,168 (13.1%)	1,02,162 (12.2%)	99,738 (11.6%)	9,018 (7.8%)	9,301 (7.5%)	9,526 (7.0%)			
**Metformin**								< 0.0001	< 0.0001	< 0.0001
		86,482 (11.1%)	99,028 (11.8%)	1,08,846 (12.7%)	7,684 (6.7%)	8,865 (7.2%)	11,062 (8.1%)			
**SUs**								< 0.0001	< 0.0001	< 0.0001
		27,778 (3.6%)	26,486 (3.2%)	24,112 (2.8%)	2,641 (2.3%)	2,428 (2%)	2,333 (1.7%)			
**Premixed insulin**								< 0.0001	< 0.0001	< 0.0001
		40,765 (5.2%)	35,220 (4.2%)	29,973 (3.5%)	4,838 (4.2%)	4,278 (3.5%)	4,165 (3.1%)			
**DPP-4i**									< 0.0001	< 0.0001
		-	2256 (0.3%)	6118 (0.7%)		243 (0.2%)	744 (0.5%)			
**Glinides**								< 0.0001	< 0.0001	< 0.0001
		8903 (1.1%)	7819 (0.9%)	6741 (0.8%)	1079 (0.9%)	910 (0.7%)	885 (0.6%)			
**Oral combination therapy**								< 0.0001	< 0.0001	< 0.0001
		3,03,125 (38.8%)	3,52,095 (42.1%)	3,86,510 (44.9%)	53,684 (46.5%)	61,813 (50%)	71,615 (52.6%)			
**AGIs**+ **metformin**								< 0.0001	< 0.0001	< 0.0001
		61,805 (7.9%)	74,791 (8.9%)	83,387 (9.7%)	8,090 (7%)	9,591 (7.8%)	11,090 (8.1%)			
**AGIs** + **SUs**								< 0.0001	< 0.0001	< 0.0001
		*46,194 (5.9%)*	*44,396 (5.3%)*	*40,622 (4.7%)*	*6,252 (5.4%)*	*6,022 (4.9%)*	*5,592 (*4.1%*)*			
**Metformin**+ **SUs**								< 0.0001	< 0.0001	< 0.0001
		41,254 (5.3%)	44,867 (5.4%)	45,167 (5.3%)	5,197 (4.5%)	5,533 (4.5%)	5,867 (4.3%)			
**AGIs** + **metformin**+ **SUs**								< 0.0001	< 0.0001	< 0.0001
		38,487 (4.9%)	44,222 (5.3%)	45,806 (5.3%)	7,026 (6.1%)	7,804 (6.3%)	8,401 (6.2%)			
**Metformin**+**DPP-4i**									0.7136	0.9439
			3982 (0.5%)	11004 (1.3%)		583 (0.5%)	1,753 (1.3%)			
**Metformin**+ **glinides**								0.5695	0.8684	0.1211
		10,642 (1.4%)	10,389 (1.2%)	9,613 (1.1%)	1,610 (1.4%)	1,539 (1.2%)	1,464 (1.1%)			
**AGIs** + **glinides**								0.4139	0.869	0.2478
		9,059 (1.2%)	8,486 (1%)	7,468 (0.9%)	1,382 (1.2%)	1,269 (1%)	1,145 (0.8%)			
**Oral**+ **insulin**								< 0.0001	< 0.0001	< 0.0001
		1,30,551 (16.7%)	1,47,566 (17.7%)	1,50,425 (17.5%)	33,861 (29.3%)	36,629 (29.6%)	39,869 (29.3%)			
**AGIs** + **premixed insulin**								< 0.0001	< 0.0001	< 0.0001
		28,454 (3.6%)	28,471 (3.4%)	25,244 (2.9%)	5,593 (4.8%)	5,006 (4.1%)	4,656 (3.4%)			
**AGIs** + **metformin**+ **insulin**								< 0.0001	< 0.0001	< 0.0001
		14,015 (1.8%)	18,503 (2.2%)	18,797 (2.2%)	4,009 (3.5%)	4,408 (3.6%)	4,599 (3.4%)			
**Metformin**+ **premixed insulin**								< 0.0001	< 0.0001	< 0.0001
		15,021 (1.9%)	16,062 (1.9%)	15,283 (1.8%)	3,386 (2.9%)	3,493 (2.8%)	3,570 (2.6%)			

AGIs, alpha-glucosidase inhibitors; Sus, sulfonylureas; DDP-4i, dipeptidyl peptidase-4 inhibitor; DPN, diabetic peripheral neuropathy.

### Changes in anti-diabetic drugs and costs

**Figure 2:**
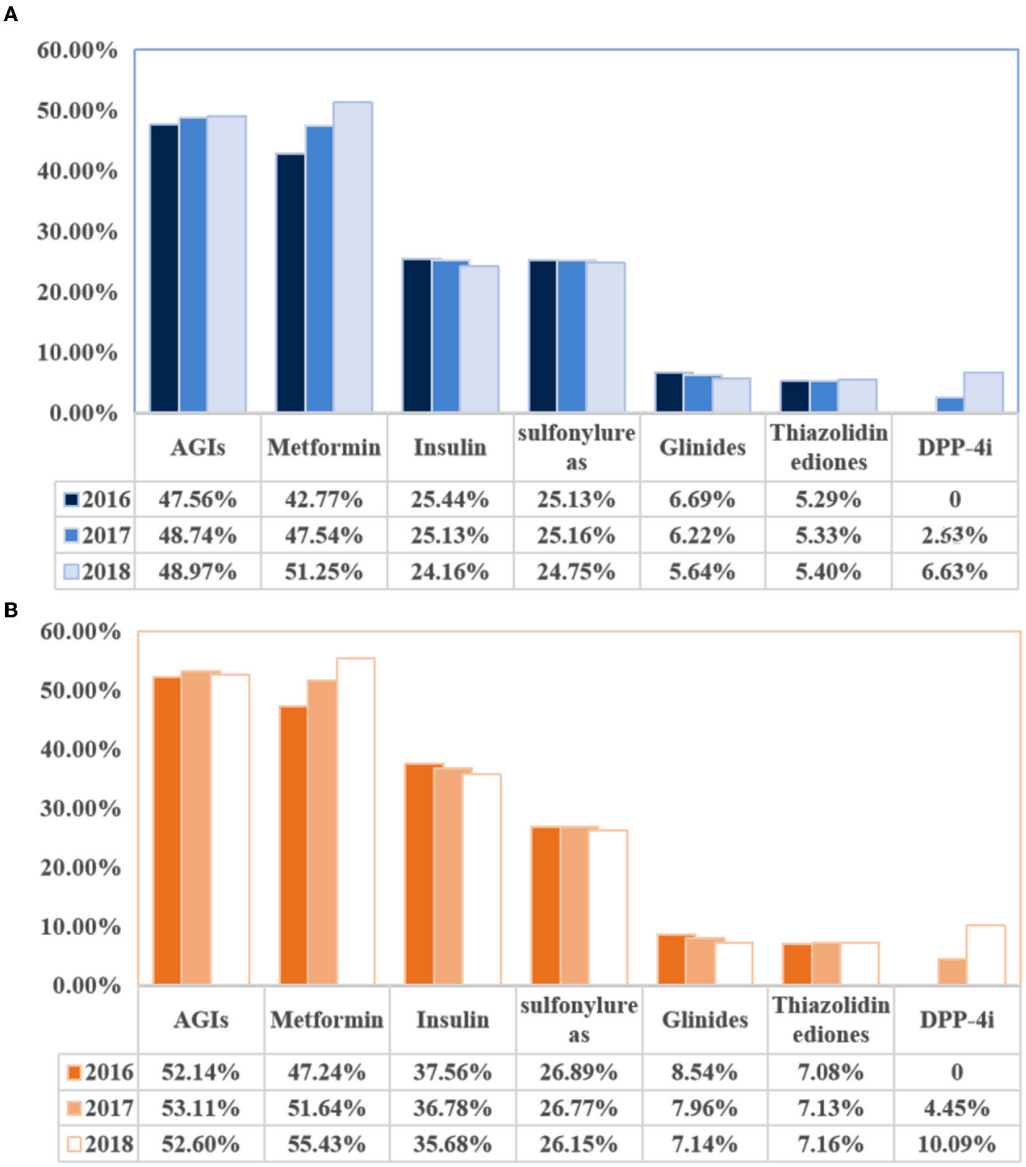
Changes in use of hypoglycemic drugs in diabetic patients with or without diabetic peripheral neuropathy. **(A)** Changes in the use of hypoglycemic drugs (non-diabetic peripheral neuropathy). **(B)** Changes in the use of hypoglycemic drugs (diabetic peripheral neuropathy). AGIs, a-glucosidase inhibitors.

Based on Figure 2, we visualized that the most commonly used OADs for diabetic patients with or without DPN were AGIs and metformin. Specifically, in 2018, 52.6% of DPN patients took AGIs, and 55.43% of DPN patients took metformin, whereas 48.97% of non-DPN patients took AGIs, and 51.25% of non-DPN patients took metformin. From 2016 to 2018, the use of metformin increased year-on-year in both patient categories. Specifically, it increased from 42.77 to 51.25% in non-DPN patients and from 47.24 to 55.43% in DPN patients. In addition, dipeptidyl peptidase-4 inhibitors (DDP4is) usage increased significantly from 2017 to 2018: from 2.55 to 6.63% in non-DPN patients and from 4.45 to 10.09% in DPN patients.

When focused on the change in medication costs over these 3 years, we found that the total annual cost/drug decreased from ¥10609.56 ±1 0697.11 to ¥9810.91 ± 9234.14 (hypoglycemic drugs decreased from ¥5333.88 ± 7757.33 to ¥5138.49 ± 6791.55 and non-hypoglycemic drugs decreased from ¥5275.68 ± 6525.5 to ¥4672.43 ± 5547.26). From 2016 to 2018, the cost per drug decreased from ¥2821.75 ± 3016.4 to ¥2520.95 ± 2509.1 (cost/anti-glycemic drug decreased from ¥2900.98 ± 3819.06 to ¥2634.51 ± 3216.73; cost/non–anti-glycemic drug decreased from ¥1928.28 ± 2303.26 to 1716.11 ± 1919.47) (Table 2).

## Discussion

In this observational study, we analyzed and compared the baseline characteristics, costs, and drugs of diabetic patients with and without DPN. We found that DPN patients were more likely to be female, to have comorbidities and diabetic complications, to use more medications, and to spend more in terms of total annual medication costs. This study also showed the change in drug and medication costs from 2016 to 2018 for diabetic patients with and without DPN.

Diabetes-related complications were key cost drivers in diabetes management (19). Several studies have analyzed the medical costs of diabetes-related complications in Asian countries (19–24). However, these studies did not distinguish between specific complications and did not look at the medication and financial burden of DPN on people with diabetes. Our study examined diabetic patients in the database of Beijing Medical Insurance to determine if DPN had an impact on their medication, as well as its financial burden for diabetic patients.

Being female was considered a risk factor for macrovascular complications of diabetes, but studies on gender differences in diabetes-related microvascular complications are scarce, and the results have been inconsistent (25). In this study, we found that the majority of diabetic patients were men, but women with diabetes were more likely to develop DPN. This result was consistent with earlier findings reported in the literature (26–29). In addition, some studies found that women with diabetes also experienced neuropathic pain more often than men (17, 28, 29). In contrast, Abraham et al. concluded that although nerve damage and polyneuropathy were more common in men with diabetes, women with diabetes had a higher frequency and intensity of pain (28). Moreover, a review by Maric-Bilkan (30) showed that the prevalence of DPN in T2DM was 46.2% in Caucasian women and 52.6% in Caucasian men; the prevalence of DPN in T2DM is higher in women than in men in Asian populations. As for T1DM, men were more likely to develop DPN. However, some studies have suggested that DPN is more prevalent in men with diabetes (25, 31). The TODAY study included 699 adolescents with T2DM and followed up with 674 of them for up to 15 years. It found a higher cumulative incidence of DPN via the Michigan Neuropathy Screening Instrument exam (MNSI-exam) and a monofilament exam in men than in women (38.5 vs. 27.2% for MNSI, *p* = 0.002; 14.0 vs. 5.1% for monofilament, *p* = 0.002) (31).

As mentioned above, there may be ethnic and/or environmental components underlying sex differences in the prevalence of DPN. In addition, female patients might be more likely to talk about neurological symptoms than male patients. Furthermore, the age of the patients who were included in the different studies also varied. Although some progress has been made in understanding the role of gender in DPN complications, many questions and controversies remain. The correlation between changes in sex hormone levels and microvascular complications has not been confirmed yet. Future research could focus on the pathophysiological mechanisms that might contribute to personalized and gender-specific treatment.

We also found that participants with DPN had a higher prevalence of comorbidities compared to non-DPN participants. It was suggested that some cardiovascular risk factors are associated with DPN, especially high triglyceride levels (14, 15, 27). However, it was debated whether dyslipidemia itself was an independent risk factor for DPN or whether hyperglycemia caused by dyslipidemia led to DPN (15). In addition, hypertension has been considered a potential risk factor for DPN (14, 32). One study found that the risk of DPN from hypertension was not related to illness duration or other complications, including microalbuminuria and overt nephropathy (32). Furthermore, osteoporosis was closely associated with DPN. A meta-analysis of 11 studies showed that patients with diabetic neuropathy were significantly more likely to develop osteoporosis or fragility fractures (overall OR 2.20, 95% CI 1.71–2.83) (12). It was also found that COPD was associated with peripheral neuropathy (33).

Similarly, in our study, diabetic patients with DPN had higher rates of DKD, DR, and DA compared to diabetic patients without DPN. DKD refers to chronic kidney disease (CKD) caused by diabetes. CKD leads to systemic vascular diseases, including microvascular disease, which are believed to contribute to more rapid nerve damage occurring in patients (34, 35). There are also functional studies that showed that both DR and DPN are closely associated with impaired microvascular reactivity (36). Previous studies reported an association between significant signs of DR and an increased risk of DPN (36–38). In addition to microangiopathy, previous studies also found that macroangiopathy is closely associated with the development of DPN (38, 39).

According to the results of this study, the total cost of medication was higher for DPN patients than for non-DPN patients. The study did not distinguish between direct and indirect medical costs and only considered the recorded total costs (direct costs are those attributable to diabetes, while indirect costs include increased absenteeism, reduced productivity, and inability to work due to disability) (40). This was consistent with other studies in which the average number of medications and the associated costs increased in patients with diabetes with complications compared to those without complications (40–43). In 2012, the economic burden of diabetes-related health resources and productivity in the United States was US $245 billion, of which 18% was spent on treating diabetes-related complications (43). In addition, several studies in China confirmed that diabetes-related complications increase the financial burden of people with diabetes. A study was conducted between 2016 and 2018 that included 18,59,039 patients with T2DM with comorbidities from the Beijing Medical Claim Data for Employees database. The study estimated the cost of complications using a generalized estimating equation model adjusted for age, gender, and the incidence of various complications. It also estimated an average total cost of US $1,712,000 for patients with combined complications of diabetes and an additional cost of US $883.90 for patients with diabetic neuropathy due to neuropathy 1 year after the onset of the disease (44).

The most frequently used OADs for diabetic patients are metformin and AGIs. Although metformin is the first-choice drug for monotherapy, T2DM patients in China tend to have post-prandial hypoglycemia, so AGIs remain the first glucose-lowering drug considered by Chinese diabetes patients (42). In this study, we found that patients with diabetes were more likely to choose premixed insulin. We assume that the main reason for this is the ease of administration and the high compliance of patients with premixed insulin. In addition to this, we believe that Chinese people have a more carbohydrate-based diet with a richer lunch and dinner, which leads to higher post-prandial blood glucose levels, and are, therefore, more suitable for premixed insulin. Patients with DPN used short-acting and long-acting insulins more frequently than non-DPN patients. We consider that DPN patients have a long duration of disease, poor glycemic control, and poor islet function and that they need better glycemic control. It has been suggested that intensive insulin therapy can improve diabetic microvascular complications, including DPN (45–47). Therefore, clinically, intensive insulin therapy regimens may be recommended more often for patients with DPN. However, data on hard endpoint outcomes for a direct comparison of basal and premixed analogs for the treatment of patients with diabetes are not yet available (48).

DPP4i was admitted to the Beijing Medical Insurance catalog in China in 2017. In this study, we saw a gradual increase in the use of DPP4i from 2017 to 2018. The usage of DPP4i was higher in DPN patients than in non-DPN patients. DPP4i benefitted from being highly orally available, posing a low risk of hypoglycemia and minimal risk of major adverse cardiovascular events. These features combined made DPP4i suitable for use in older adult or vulnerable patients who often suffered from comorbidities and complications (49). Animal studies verified that DPP4i improved neuropathy in streptozotocin (STZ)-induced diabetic mice (50, 51). However, population studies on the effects of DPP4i on DPN were scarce. Ashit et al. conducted a prospective, open study including 20 cases of T2DM. The study assessed the efficacy and tolerability of DPP4i in DPN and diabetic autonomic neuropathy. They concluded that Teneligliptin not only improved patients' glycemic status but also improved their peripheral and autonomic neuropathy and reduced vascular inflammation (52).

There is a lack of studies addressing the burden of a single complication of diabetes, and there are even fewer studies addressing the burden of DPN. This is the first study in China to compare the burden of DPN and non-DPN patients. This study, thus, has significant value. Its data, stemming from a large sample, adequately reflects the differences between DPN and non-DPN patients in terms of baseline, medication use, and medication costs and provides very important reference values for understanding DPN, medication use, and the resulting socio-economic burden.

## Limitations

Our study is not without limitations. First, this study is an observational cross-sectional study, reflecting data for each year from 2016 to 2018 for diabetic patients in Beijing only, without following up with patients, so we cannot make statements regarding changes in the same patients' conditions, medication, and medication costs over time. Later studies can examine patients with 3 years of follow-up for further analysis. In addition, we did not stratify type 1 and type 2 diabetes mellitus. Considering the different pathogenesis of DPN in type 1 and type 2 diabetes mellitus, there may be some differences in comorbidities, complications, and medication use. Meanwhile, this study only reflects data from Beijing, which has a developed economy, and the burden of disease in China varies greatly by geographical location, so the data in this study cannot reflect the national situation. Moreover, data on newer hypoglycemic drugs were lacking, and the use of new hypoglycemic drugs and potentially beneficial effects may improve the illness and financial burden associated with DPN. Finally, this study lacked data on economic factors, education, income, etc., which might also have an impact on medication choice and expenditure.

## Conclusion

In conclusion, this retrospective study, based on a large sample of diabetic patients diagnosed in outpatient clinics with Beijing Health Insurance, analyzed the medication regimens and costs of treatment for diabetic patients with DPN and those without DPN. DPN is more common in female diabetic patients, and they are more likely to have comorbidities and diabetic complications, use more medications, and have higher total medical costs. Our research contributes to the literature on the growing global economic burden of DPN. Knowing the cost of diabetes and its major complications is essential for raising awareness and devising strategies to reduce its prevalence and impact, as well as for early screening and standardized treatment that can reduce costs.

## Data availability statement

The original contributions presented in the study are included in the article/supplementary material, further inquiries can be directed to feisijia0924@163.com.

## Ethics statement

Written informed consent was obtained from the individual(s) for the publication of any potentially identifiable images or data included in this article.

## Author contributions

LG, QP, and SF designed the study and wrote the first draft of the manuscript. JL, HC, and WW analyzed and interpreted the data. QP, SF, and LZ devised the whole project and critically reviewed the manuscript. FX and LG revised the manuscript. All authors read and approved the final manuscript.

## References

[1] Zimmet PZ Magliano DJ Herman WH Shaw JE. Diabetes: a 21st century challenge. Lancet Diabetes Endocrinol. (2014) 2:56–64. 10.1016/S2213-8587(13)70112-8 24622669

[2] International Diabetes Federation. Idf Diabetes Atlas. 10th ed. Brussels, Belgium (2021). Available online at: http://www.Diabetesatlas.Org (accessed February 10, 2023).

[3] Harding JL Pavkov ME Magliano DJ Shaw JE Gregg EW. Global trends in diabetes complications: a review of current evidence. Diabetologia. (2019) 62:3–16. 10.1007/s00125-018-4711-2 30171279

[4] Rosa MQM Rosa RDS Correia MG Araujo DV Bahia LR Toscano CM. Disease and economic burden of hospitalizations attributable to diabetes mellitus and its complications: a nationwide study in Brazil. Int J Environ Res Public Health. (2018) 15:294. 10.3390/ijerph15020294 29419786 PMC5858363

[5] Bommer C Heesemann E Sagalova V Manne-Goehler J Atun R Barnighausen T. The global economic burden of diabetes in adults aged 20–79 years: a cost-of-illness study. Lancet Diabetes Endocrinol. (2017) 5:423–30. 10.1016/S2213-8587(17)30097-9 28456416

[6] Bao X Yang C Fang K Shi M Yu G Hu Y. Hospitalization costs and complications in hospitalized patients with type 2 diabetes mellitus in Beijing, China. J Diabetes. (2017) 9:405–11. 10.1111/1753-0407.12428 27194641

[7] Wu H Eggleston KN Zhong J Hu R Wang C Xie K. How do type 2 diabetes mellitus (T2dm)-related complications and socioeconomic factors impact direct medical costs? A cross-sectional study in Rural Southeast China. BMJ Open. (2018) 8:e020647. 10.1136/bmjopen-2017-020647 30389755 PMC6224711

[8] Lin WQ Cai ZJ Chen T Liu MB Li N Zheng B. Cost-effectiveness of dipeptidylpeptidase-4 inhibitors added to metformin in patients with type 2 diabetes in China. Front Endocrinol. (2021) 12:684960. 10.3389/fendo.2021.684960 34484112 PMC8415028

[9] Yang K Wang Y Li YW Chen YG Xing N Lin HB. Progress in the treatment of diabetic peripheral neuropathy. Biomed Pharmacother. (2022) 148:112717. 10.1016/j.biopha.2022.112717 35193039

[10] Moon SS Kim CH Kang SM Kim ES Oh TJ Yun JS. Status of diabetic neuropathy in Korea: a national health insurance service-national sample cohort analysis (2006 to 2015). Diabetes Metab J. (2021) 45:115–9. 10.4093/dmj.2020.0120 33327050 PMC7850872

[11] Lixin G. Diabetes Atlas of China. Beijing: People's Medical Publishing House (2022).

[12] Liu C Lv H Niu P Tan J Ma Y. Association between diabetic neuropathy and osteoporosis in patients: a systematic review and meta-analysis. Arch Osteoporos. (2020) 15:125. 10.1007/s11657-020-00804-6 32779030

[13] De Visser A Hemming A Yang C Zaver S Dhaliwal R Jawed Z. The adjuvant effect of hypertension upon diabetic peripheral neuropathy in experimental type 2 diabetes. Neurobiol Dis. (2014) 62:18–30. 10.1016/j.nbd.2013.07.019 23938761

[14] Tesfaye S Chaturvedi N Eaton SE Ward JD Manes C Ionescu-Tirgoviste C. Vascular risk factors and diabetic neuropathy. N Engl J Med. (2005) 352:341–50. 10.1056/NEJMoa032782 15673800

[15] Jende JME Groener JB Oikonomou D Heiland S Kopf S Pham M. Diabetic neuropathy differs between type 1 and type 2 diabetes: insights from magnetic resonance neurography. Ann Neurol. (2018) 83:588–98. 10.1002/ana.25182 29443416

[16] Iqbal Z Azmi S Yadav R Ferdousi M Kumar M Cuthbertson DJ. Diabetic peripheral neuropathy: epidemiology, diagnosis, and pharmacotherapy. Clin Ther. (2018) 40:828–49. 10.1016/j.clinthera.2018.04.001 29709457

[17] Candrilli SD Davis KL Kan HJ Lucero MA Rousculp MD. Prevalence and the associated burden of illness of symptoms of diabetic peripheral neuropathy and diabetic retinopathy. J Diabetes Complications. (2007) 21:306–14. 10.1016/j.jdiacomp.2006.08.002 17825755

[18] Selvarajah D Kar D Khunti K Davies MJ Scott AR Walker J. Diabetic peripheral neuropathy: advances in diagnosis and strategies for screening and early intervention. Lancet Diabetes Endocrinol. (2019) 7:938–48. 10.1016/S2213-8587(19)30081-6 31624024

[19] Jiao F Wong CKH Tang SCW Fung CSC Tan KCB McGhee S. Annual direct medical costs associated with diabetes-related complications in the event year and in subsequent years in Hong Kong. Diabet Med. (2017) 34:1276–83. 10.1111/dme.13416 28636749

[20] Shuyu Ng C Toh MP Ko Y Yu-Chia Lee J. Direct medical cost of type 2 diabetes in Singapore. PLoS ONE. (2015) 10:e0122795. 10.1371/journal.pone.0122795 25816299 PMC4376523

[21] Wong CKH Jiao F Tang EHM Tong T Thokala P Lam CLK. Direct medical costs of diabetes mellitus in the year of mortality and year preceding the year of mortality. Diabetes Obes Metab. (2018) 20:1470–8. 10.1111/dom.13253 29430799

[22] Chen HY Kuo S Su PF Wu JS Ou HT. Health care costs associated with macrovascular, microvascular, and metabolic complications of type 2 diabetes across time: estimates from a population-based cohort of more than 0.8 million individuals with up to 15 years of follow-up. Diabetes Care. (2020) 43:1732–40. 10.2337/dc20-0072 32444454 PMC7372047

[23] Cheng S-W Wang C-Y Chen J-H Ko Y. Healthcare costs and utilization of diabetes-related complications in Taiwan. Medicine. (2018) 97:e11602. 10.1097/MD.0000000000011602 30075532 PMC6081128

[24] Kim TH Chun KH Kim HJ Han SJ Kim DJ Kwak J. Direct medical costs for patients with type 2 diabetes and related complications: a prospective cohort study based on the korean national diabetes program. J Korean Med Sci. (2012) 27:876–82. 10.3346/jkms.2012.27.8.876 22876053 PMC3410234

[25] de Ritter R Sep SJS van der Kallen CJH van Greevenbroek MMJ de Jong M Vos RC. Sex differences in the association of prediabetes and type 2 diabetes with microvascular complications and function: the Maastricht study. Cardiovasc Diabetol. (2021) 20:102. 10.1186/s12933-021-01290-x 33962619 PMC8106227

[26] Liu Z Fu C Wang W Xu B. Prevalence of chronic complications of type 2 diabetes mellitus in outpatients - a cross-sectional hospital based survey in Urban China. Health Qual Life Outcomes. (2010) 8:62. 10.1186/1477-7525-8-62 20579389 PMC2906445

[27] Mizokami-Stout KR Li Z Foster NC Shah V Aleppo G McGill JB. The contemporary prevalence of diabetic neuropathy in type 1 diabetes: findings from the T1d exchange. Diabetes Care. (2020) 43:806–12. 10.2337/dci20-0028 32029635 PMC7085805

[28] Abraham A Barnett C Katzberg HD Lovblom LE Perkins BA Bril V. Sex differences in neuropathic pain intensity in diabetes. J Neurol Sci. (2018) 388:103–6. 10.1016/j.jns.2018.03.008 29627001

[29] Alkhatatbeh M Abdul-Razzak KK. Neuropathic pain is not associated with serum vitamin D but is associated with female gender in patients with type 2 diabetes mellitus. BMJ Open Diabetes Res Care. (2019) 7:e000690. 10.1136/bmjdrc-2019-000690 31275577 PMC6577304

[30] Maric-Bilkan C. Sex differences in micro- and macro-vascular complications of diabetes mellitus. Clin Sci. (2017) 131:833–46. 10.1042/CS20160998 28424377

[31] Group TS. Risk factors for diabetic peripheral neuropathy in adolescents and young adults with type 2 diabetes: results from the today study. Diabetes Care. (2021) 45:1065–72. 10.2337/dc21-1074 34716210 PMC9174958

[32] Forrest KY Maser RE Pambianco G. Hypertension as a risk factor for diabetic neuropathy: a prospective study. Diabetes. (1997) 46:665–70. 10.2337/diabetes.46.4.665 9075809

[33] Kahnert K Föhrenbach M Lucke T Alter P Trudzinski FT Bals R. The impact of COPD on polyneuropathy: results from the german COPD cohort cosyconet. Respir Res. (2020) 21:28. 10.1186/s12931-020-1293-6 31959163 PMC6971882

[34] Yang Z Lou X Zhang J Nie R Liu J Tu P. Association between early markers of renal injury and type 2 diabetic peripheral neuropathy. Diabetes Metab Syndr Obes. (2021) 14:4391–7. 10.2147/DMSO.S335283 34744444 PMC8565989

[35] Liu J Yuan X Liu J Yuan G Sun Y Zhang D. Risk factors for diabetic peripheral neuropathy, peripheral artery disease, and foot deformity among the population with diabetes in Beijing, China: a multicenter, cross-sectional study. Front Endocrinol. (2022) 13:824215. 10.3389/fendo.2022.824215 35733764 PMC9207340

[36] Ding J Cheung CY Ikram MK Zheng YF Cheng CY Lamoureux EL. Early retinal arteriolar changes and peripheral neuropathy in diabetes. Diabetes Care. (2012) 35:1098–104. 10.2337/dc11-1341 22374638 PMC3329839

[37] Pan Q Li Q Deng W Zhao D Qi L Huang W. Prevalence of and risk factors for peripheral neuropathy in Chinese patients with diabetes: a multicenter cross-sectional study. Front Endocrinol. (2018) 9:617. 10.3389/fendo.2018.00617 30455667 PMC6230581

[38] Khawaja N Abu-Shennar J Saleh M Dahbour SS Khader YS Ajlouni KM. The prevalence and risk factors of peripheral neuropathy among patients with type 2 diabetes mellitus; the case of Jordan. Diabetol Metab Syndr. (2018) 10:8. 10.1186/s13098-018-0309-6 29483946 PMC5822644

[39] Valensi P Giroux C Seeboth-Ghalayini B Attali J-R. Diabetic peripheral neuropathy: effects of age, duration of diabetes, glycemic control, and vascular factors. J Diabetes Complications. (1997) 11:27–34. 10.1016/S1056-8727(95)00086-0 9025010

[40] American Diabetes Association. Economic costs of diabetes in the U.S. in 2017. Diabetes Care. (2018) 41:917–28. 10.2337/dci18-0007 29567642 PMC5911784

[41] Li R Bilik D Brown MB Zhang P Ettner SL Ackermann RT. Medical costs associated with type 2 diabetes complications and comorbidities. Am J Manag Care. (2013) 19:421–30. 23781894 PMC4337403

[42] Guo L Zheng J Pan Q Zhang Q Zhou Y Wang W. Changes in direct medical cost and medications for managing diabetes in Beijing, China, 2016 to 2018: electronic insurance data analysis. Ann Fam Med. (2021) 19:332–41. 10.1370/afm.2686 34264834 PMC8282298

[43] American Diabetes Association. Economic costs of diabetes in the U.S. in 2012. Diabetes Care. (2013) 36:1033–46. 10.2337/dc12-2625 23468086 PMC3609540

[44] Wu JH Wu Y Wang ZJ Wu YQ Wu T Wang MY. Healthcare costs associated with complications in patients with type 2 diabetes among 1.85 million adults in Beijing, China. Int J Environ Res Public Health. (2021) 18:3693. 10.3390/ijerph18073693 33916217 PMC8036594

[45] Nathan DM Buse JB Davidson MB Ferrannini E Holman RR Sherwin R. Medical management of hyperglycaemia in type 2 diabetes mellitus: a consensus algorithm for the initiation and adjustment of therapy: a consensus statement from the American Diabetes Association and the European Association for the Study of Diabetes. Diabetologia. (2009) 52:17–30. 10.1007/s00125-008-1157-y 18941734

[46] Ohkubo Y Kishikawa H Araki E Miyata T Isami S Motoyoshi S. Intensive insulin therapy prvents the progression of diabetic microvascular complicaions in Japanese patients with non insulin dependent diabetes mellitus a randomized prospective 6 year study. Diab Res Clin Pract. 28:103–17. 10.1016/0168-8227(95)01064-K 7587918

[47] ElSayed NA Aleppo G Aroda VR Bannuru RR Brown FM Bruemmer D. 12. Retinopathy, neuropathy, and foot care: standards of care in diabetes-2023. Diabetes Care. (2023) 46:S203–15. 10.2337/dc23-S012 36507636 PMC9810462

[48] Vaag A Lund SS. Insulin initiation in patients with type 2 diabetes mellitus: treatment guidelines, clinical evidence and patterns of use of basal vs premixed insulin analogues. Eur J Endocrinol. (2012) 166:159–70. 10.1530/EJE-11-0022 21930715 PMC3260696

[49] Deacon CF. Dipeptidyl peptidase 4 inhibitors in the treatment of type 2 diabetes mellitus. Nat Rev Endocrinol. (2020) 16:642–53. 10.1038/s41574-020-0399-8 32929230

[50] Tsuboi K Mizukami H Inaba W Baba M Yagihashi S. The dipeptidyl peptidase Iv inhibitor vildagliptin suppresses development of neuropathy in diabetic rodents: effects on peripheral sensory nerve function, structure and molecular changes. J Neurochem. (2016) 136:859–70. 10.1111/jnc.13439 26603140

[51] Bianchi R Cervellini I Porretta-Serapiglia C Oggioni N Burkey B Ghezzi P. Beneficial effects of Pkf275–055, a novel, selective, orally bioavailable, long-acting dipeptidyl peptidase iv inhibitor in streptozotocin-induced diabetic peripheral neuropathy. J Pharmacol Exp Ther. (2012) 340:64–72. 10.1124/jpet.111.181529 21984837

[52] Syngle A Chahal S Vohra K. Efficacy and tolerability of Dpp4 inhibitor, teneligliptin, on autonomic and peripheral neuropathy in type 2 diabetes: an open label, pilot study. Neurol Sci. (2021) 42:1429–36. 10.1007/s10072-020-04681-2 32803534

